# Identification of Potential Genomic Alterations and the circRNA-miRNA-mRNA Regulatory Network in Primary and Recurrent Synovial Sarcomas

**DOI:** 10.3389/fmolb.2021.707151

**Published:** 2021-08-13

**Authors:** Qing Yao, Yong-Lai He, Ning Wang, Shuang-Shuang Dong, Mei Er Tu He Ta Mi Shi, Xiao Feng, Hao Chen, Li-Juan Pang, Hong Zou, Wen-Hu Zhou, Feng Li, Yan Qi

**Affiliations:** ^1^ Department of Pathology, Shihezi University School of Medicine and the First Affiliated Hospital to Shihezi University School of Medicine, Shihezi, China; ^2^ Department of Pathology, Certral People’s Hospital of Zhanjiang and Zhanjiang Central Hospital, Guangdong Medical University, Zhanjiang, China; ^3^ Xiangya School of Pharmaceutical Sciences, Central South University, Changsha, China; ^4^ Department of Pathology, Beijing Chaoyang Hospital, Capital Medical University, Beijing, China

**Keywords:** genome-wide SNP analysis, circRNA, ceRNA, gene, primary synovial sarcoma, recurrent synovial sarcoma

## Abstract

**Introduction:** Synovial sarcoma (SS) is one of the most invasive soft tissue sarcomas, prone to recurrence and metastasis, and the efficacy of surgical treatment and chemotherapy for SS remains poor. Therefore, the diagnosis and treatment of SS remain a significant challenge. This study aimed to analyze the mutated genes of primary SS (PSS) and recurrent SS (RSS), discover whether these sarcomas exhibit some potential mutated genes, and then predict associated microRNAs (miRNA) and circular RNAs (circRNA) by analyzing the mutated genes. We focused on the regulation mechanism of the circRNA-miRNA-mutated hub gene in PSS and RSS.

**Methods:** We performed a comprehensive genomic analysis of four pairs of formalin-fixed paraffin-embedded samples of PSS and RSS, using Illumina human exon microarrays. The gene ontology (GO), Kyoto Encyclopedia of Genes and Genomes (KEGG) function, and pathway enrichment of the mutated genes were analyzed, and the protein-protein interaction (PPI) network was forecast using String software 11.0. The hub genes were then obtained using the Molecular Complex Detection (MCODE) plug-in for Cytoscape 3.7.2 and were used to analyze overall survival (OS) using the Gene Expression Profiling Interactive Analysis (GEPIA) database. The corresponding miRNAs were obtained from the miRDB 5.0 and TargetScan 7.2 databases. The corresponding circRNAs of the hub genes were found through the miRNAs from these databases: Circbank, CircInteractome, and StarBase v2.0. Thereafter we set up a competing endogenous RNA (ceRNA) network with circRNA-miRNA and miRNA-messenger RNA (mRNA) pairs.

**Results:** Using the chi-squared test, 391 mutated genes were screened using a significance level of *p*-values < 0.01 from the four pairs of PSS and RSS samples. A GO pathway analysis of 391 mutated genes demonstrated that differential expression mRNAs (DEmRNAs) might be bound up with the “positive regulation of neurogenesis,” “cell growth,” “axon part,” “cell−substrate junction,” or “protein phosphatase binding” of SS. The PPI network was constructed using 391 mutated genes, and 53 hub genes were identified (*p* < 0.05). Eight variant hub genes were discovered to be statistically significant using the OS analysis (*p* < 0.05). The circRNA-miRNA-mRNA (ceRNA) network was constructed, and it identified two circRNAs (hsa_circ_0070557 and hsa_circ_0070558), 10 miRNAs (hsa-let-7a-3p, hsa-let-7b-3p, hsa-let-7f-1-3p, hsa-let-7f-2-3p, hsa-mir-1244, hsa-mir-1197, hsa-mir-124-3p, hsa-mir-1249-5p, hsa-mir-1253, and hsa-mir-1271-5p) and five hub genes (CENPE, ENPP3, GPR18, MDC1, and PLOD2).

**Conclusion:** This study screened novel biological markers and investigated the differentiated circRNA-miRNA-mutated hub gene axis, which may play a pivotal role in the nosogenesis of PSS and RSS. Some circRNAs may be deemed new diagnostic or therapeutic targets that could be conducive to the future clinical treatment of SS.

## Introduction

Synovial sarcoma (SS) is a highly invasive soft tissue sarcoma (STS) that predominantly occurs in the limbs of adults in their 30s and accounts for about 5–10% of all STSs (Riedel et al., 2018; Hale et al., 2019). Its pathogenic characteristics are related to the unique chromosomal translocation t (X; 18) (p11.2; q11.2), leading to the formation and expression of the oncogenic fusion gene SS18-SSX (Riggi et al., 2018). Accepted standard treatment methods for SS include extensive surgical resection and chemotherapy. However, treatment has not been shown to significantly improve SS outcomes (Yoshimatsu et al., 2020), which were closely related to the occurrence of metastasis or local recurrence in more than half of patients, and spread easily to sites, such as the lungs and bone (El Beaino et al., 2017). In recent years, some studies have shown that different genomic alterations were found in primary and recurrent tumors, including chromatin mutation, RNA splicing, and epigenomic regulation. However, information on the underlying molecular mechanisms of aggressive SS is still lacking. Much work remains to be done to explore the different genomic alterations and investigate the natural history of primary SS (PSS) and recurrent SS (RSS). This could help guide the development of good clinical therapies for SS.

Qi et al. (2015) reported that genome-wide single nucleotide polymorphisms (SNPs) are used to assess gene functions because SNPs can use limited sample material to study DNA sequence variations occurring in the genome and transcriptome and identify their pathogenic genes (Guo and Rotter, 2019). Therefore, therapy can be targeted based on mutated genes in PSS and RSS.

In recent years, an increasing number of studies have shown that genes and microRNA (miRNA) can be regulated by circular RNA (circRNA) (Zeng et al., 2020) and that the aberrant expression of circRNA and miRNA contributes to human diseases, including cancer (Lin et al., 2020; Sarkar et al., 2020). With the emergence of high-throughput RNA sequencing and next-generation sequencing technology, circRNA has been increasingly studied (Lu and Thum, 2019). Liu et al. (2015)(Kristensen et al., 2019) found that, compared to their linear counterparts, circRNAs are highly stable because of their covalently closed structure, are less susceptible to degradation by enzymes, and can be discovered in exosomes, cell-free saliva, and plasma. Moreover, circRNAs can act as miRNA sponges to regulate gene expression (Zheng et al., 2016). Therefore, because of some features of circRNA stability, some studies have suggested that circRNAs can be used as markers for disease diagnosis and prognosis (Kristensen et al., 2019).

Thus, to provide insights into the pathogenesis of PSS and RSS, we aimed to obtain differentially expressed genes and extract hub genes from PSS and RSS, and circRNA-miRNA-messenger RNA (mRNA) competing endogenous RNA (ceRNA) networks using genome-wide SNP analysis and data from existing open-source resources.

## Materials and Methods

### Patients and Tissue Specimens

Four formalin-fixed paraffin-embedded (FFPE) samples of PSS and RSS, in which SNP expression was proven to differ between primary and recurrent samples, were acquired from patients treated at the Department of Pathology, First Affiliated Hospital, Shihezi University, School of Medicine. The study included one male and three female patients with biphasic SS. The patients' ages were 64, 22, 10, and 37 years (mean age 34 years). Histological and immunohistochemical analyses confirmed the diagnosis of SS, and the presence of a fusion gene was detected using a one-step reverse transcription-polymerase chain reaction (QIAGEN, Venlo, The Netherlands). The characteristics of the four paired PSS and RSS patients enrolled in this study are shown in Table 1. Clinical staging was performed according to the National Comprehensive Cancer Network 2016 guidelines for soft-tissue tumors (von Mehren et al., 2016).

**TABLE 1:** The basic information of the patients.

Case	Sex/age	Classification	Molecular pathological classification	Diagnosis	Location	Size (cm)	Metastases	Grade	Stages
P1	F/22	primary synovial sarcoma	SYT-SSX2	BSS	Left heel	4.5 × 3×1.5	None	III	ⅡA
R1	F/22	recurrent synovial sarcoma	SYT-SSX2	BSS	Left heel	3 × 3×2.5	None		
P2	F/10	primary synovial sarcoma	SYT-SSX1	BSS	Posterior to right elbow	5 × 4.8×3	None	III	IV
R2	F/10	recurrent synovial sarcoma	SYT-SSX	BSS		NA	Bone marrow cavity		
P3	M/64	primary synovial sarcoma	SYT-SSX	SS	Anterior tibialis of right	5.6 × 1.2×1.1	None	III	I
R3	M/70	recurrent synovial sarcoma	SYT-SSX	SS	Left hip	11.5 × 10.5×7	None		
P4	F/37	primary synovial sarcoma	SYT-SSX	SS	Left pubic ramus	3.5 × 3.3×1	None	III	NA
R4	F/37	recurrent synovial sarcoma	SYT-SSX	BSS	Left ischial tuberosity bursa	NA	None	NA	NA

P: primary; R: recurrent; BSS: Biphasic synovial sarcoma; Grade: Histopathological grade; Stages: clinical stages; NA: not available.

In this study, all patients provided informed consent, and the patients’records and information were anonymized before analysis. The study was organized following the instructions approved by the Clinical Research Ethics Committee of the First Affiliated Hospital of Shihezi University School of Medicine.

### DNA Extraction and SNP Array

Genomic DNA was isolated from four FFPE tumor samples using the QIAamp DNA Micro Kit (Qiagen Inc., Valencia, CA, United States) according to the manufacturer’s instructions. The minimum amount of genomic DNA successfully subjected to genomic SNP microarray analysis was 1 ug, with quality metrics of A260/280 = 1.7–2.0 and A260/230 > 1.6. According to the manufacturer’s recommendation, the patient’s DNA target was prepared and hybridized using an Infinium HD analysis Superchip. CEL files were imported into Illumina Human Exome-12v1.1 (Beijing Compass Biological Technology Co. Ltd.; http://www.kangpusen.com/index.html) for quality analysis according to the “Quality Control Assessment in Genotyping Console.” An association analysis of controls versus cases was performed using PLINK (http://pngu.mgh.harvard.edu/∼purcell/plink) using Fisher’s exact test, chi-squared test, and estimated odds ratios. Pearson’s chi-square values and *p-*values (chi-squared and Fisher exact tests) were estimated using Daploview 4.2 PLINK data.

### Illumina Exon Microarray Detection

A custom microarray with exon-level resolution was investigated using Illumina Human Exome-12v1.1 to identify gross deletions and duplications. The microarray contains approximately 60,000 integrated oligonucleotide probes that have been annotated against the human genome. The density of probes increased across exons and 300 bp of flanking intronic sequence. After the validation run, only the best-performing probes were selected for use in the final array design.

The specific method used is as follows: first, a DNA standard plate was prepared, followed by a QNT standard plate with diluted Pico Green and a QNT sample plate with PicoGreen and DNA. Subsequently, DNA samples were moved to the MSA1 plate to denature and neutralize them and were then used for amplification by overnight incubation. The following day, an endpoint fragmentation was used to enzymatically fragment the DNA to avoid excessive fragmentation. Therefter, the DNA samples were treated with 2-propanol and PM1 to precipitate MSA1, and RA1 was used to resuspend the precipitated DNA. Next, the resuspended DNA samples were dispensed on BeadChips, which were incubated in an Illumina Hybridization Oven for approximately 16–24 h to hybridize the samples onto them. The following day, a stained BeadChip was prepared, and nonhybridized and nonspecifically hybridized DNA was washed away. There are eight categories of internal controls per sample in an Illumina SNP microarray experiment: staining, extension, hybridization, stringency, target removal, recovery, nonspecific binding, and nonpolymorphic controls. Quality analysis of the Illumina Human Exome-12v1.1 reporter samples indicated a call rate of 0.7526.

### Gene Ontology and Kyoto Encyclopedia of Genes and Genomes Functional Enrichment Analysis

GO (Dutkowski et al., 2013) is a key tool for annotating genes and analyzing their biological processes (BPs), molecular functions (MFs), and cellular components (CCs). To further understand the enrichment of SS mutated genes and their main functional annotations, the mutated genes of SNPs were evaluated by the GO annotation and KEGG (Kanehisa et al., 2021) pathways, using cluster spectral packages in R 3.51 (geoSeq). *p-*values < 0.05 were considered statistically significant.

### Constructing a Protein-Protein Interaction Network and Finding Hub Genes of SNPs

The String online database (11.0; https://string-db.org/ ) provides reliable information on PPIs (Szklarczyk et al., 2011). In this study, PPIs with a combined score >0.9 were considered statistically significant. The PPI network was constructed through mutant genes of SNPs in the String online database. Cytoscape is an open-source software platform for visualizing molecular interaction networks and biological pathways and integrating these networks with annotations, gene expression profiles, and other state data (Shannon et al., 2003) and was used to analyze the obtained gene or protein networks (Cline et al., 2007). The Molecular Complex Detection (MCODE) is an application plug-in for Cytoscape 3.7.2, whose algorithm can detect densely connected regions in large PPI networks that may represent molecular complexes. Therefore, the MCODE plug-in should be used to identify the hub genes of SNPs obtained from the PPI network (Bandettini et al., 2012). The hub gene criteria were as follows: degree cut-off = 2, node score cut-off = 0.2, K-core = 2, Max depth = 100.

### Survival Analysis

The total survival rate of hub genes acquired from the PPI network was analyzed using the Gene Expression Profiling Interactive Analysis (GEPIA) online database. GEPIA (http://gepia.cancer-pku.cn/detail.php) (Tang et al., 2017) is a web-based tool that provides fast and customizable capabilities based on The Cancer Genome Atlas and Genotype-Tissue Expression data. GEPIA offers key interactive and customizable features, including differential expression, correlation, patient survival analyses. The log-rank test was used for statistical analysis. In this study, overall survival (OS) was analyzed by hub genes differentially expressed between SS tissues and normal tissues. The threshold for prognostic survival significance was a *p-*value < 0.05. A subnetwork was constructed based on hub genes of SNPs that were meaningful for OS.

### Predicting Binding Sites of miRNAs and circRNAs and Construction of the ceRNA Network

The TargetScan 7.2 (http://www.targetscan.org/vert_72/) and miRDB 5.0 (Chen and Wang, 2020) (http://mirdb.org/) online databases were used to predict the association between mutant genes and miRNAs. Only the miRNAs present in these two databases were regarded as candidate miRNAs. The Circbank (Liu et al., 2019) (http://www.circbank.cn/downloads.html), CircInteractome (Dudekula et al., 2016) (https://circinteractome.nia.nih.gov/index.html), and StarBase V2.0 (Li et al., 2014) (https://www.ncbi.nlm.nih.gov/geo/) online databases were used to find differentially expressed circRNAs (DEcircRNAs) targeted by miRNAs. The ceRNA regulatory network was constructed using circRNA-miRNA pairs and miRNA-mRNA pairs. Finally, the ceRNA network was acquired through Cytoscape 3.7.2.

## Results

### Exome-Chip Data and Functional Annotation of SS-Associated Variants

**FIGURE 1:**
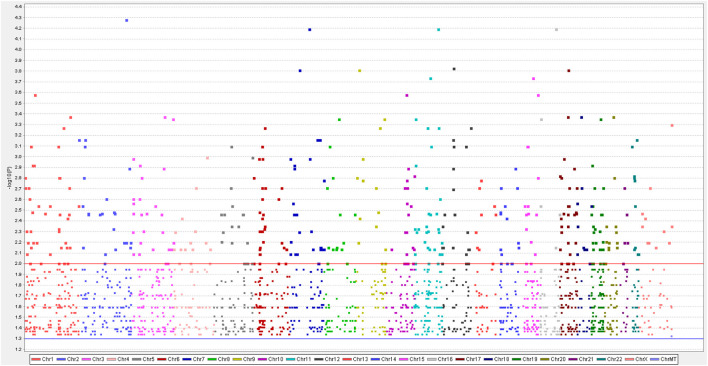
In the aggregate to 2024 single nucleotide polymorphisms (SNPs) were obtained by a genome-wide analysis of four samples of synovial sarcomas. A Manhattan plot indicates negative log-transformed *p-*values of the case-control allele frequency significance on the Y-axis. The color-coding on the X-axis represents the number of chromosomes. Gene names indicated by single dots demonstrate SNPs of greatest significance or with potential disease relevance.

**FIGURE 2:**
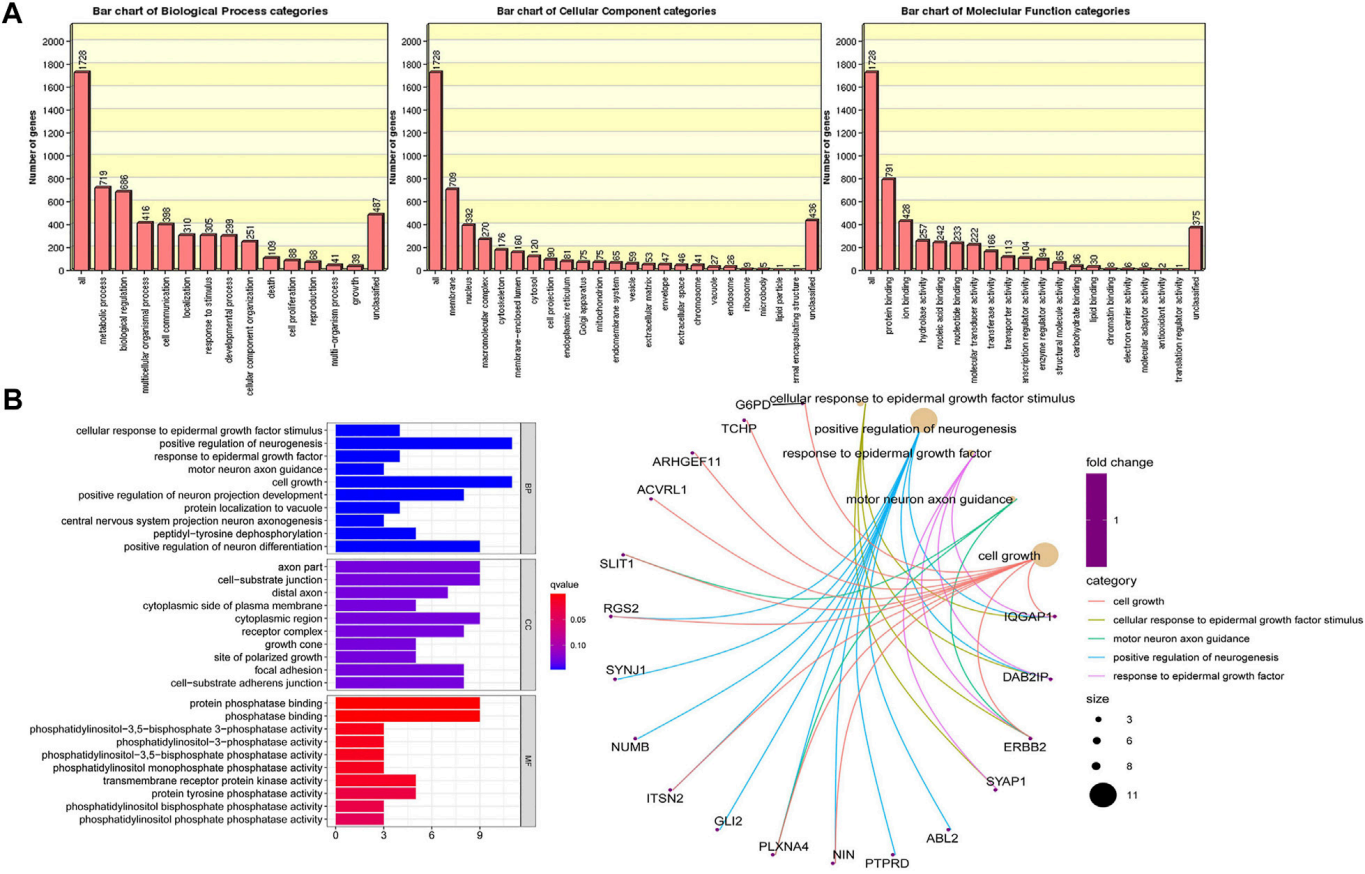
The differential single nucleotide polymorphisms (SNPs) of target genes were found to be functionally enriched by a gene ontology enrichment analysis, which mainly included three aspects: biological processes (BPs), molecular functions (MFs) and cellular components (CCs). **(A)** Functional enrichment analysis of 1828 SNP variant genes (*p* < 0.05); **(B)** Functional enrichment analysis of 391 SNP variant genes (*p* < 0.01).

We identified approximately 2024 single nucleotide variants, small insertions, and deletion alterations using Illumina Human Exome Microarrays in the primary and recurrent cases across the genomes of the four patients (Figure 1) (Supplementary Material 1). The PLINK analysis of the SNP-chip data revealed that 1828 SNPs were significant (*p* < 0.05). Further, GO analysis showed that SNPs of 1828 are mainly concentrated in “metabolic process,” “biological regulation,” “multicellular organismal process” (categorized as BPs), “membranes,” “the nucleus,” “macromolecular complexes” (categorized as CCs), “protein binding,” “ion binding,” and “hydrolase activity” (categorized as MFs) (Figure 2A). Of note, the KEGG analysis only focused on the extracellular matrix-receptor interaction (*p* < 0.05). To make our data meaningful, we selected the significant data (*p* < 0.01) for further analysis, including 391 SNPs (Supplementary Material 2). Subsequently, the result of the GO analysis indicated that 391 variant genes of SNPs were mainly concentrated on “positive regulation of neurogenesis,” “cell growth,” “positive regulation of neuron differentiation” (categorized as BPs), “axon part,” “cell−substrate junction” (categorized as CCs), “protein phosphatase binding,” and “phosphatase binding” (categorized as MFs) (Figure 2B). However, no statistically significant pathway was found by the KEGG analysis for 391 mutated genes of SNPs (*p* > 0.05). We, therefore, conclude that the genes from which we obtained mutations were associated with neuronal differentiation, and studies suggest that SSs have a possible neural origin (Ishibe et al., 2008).

### Construction of the Protein-Protein Interaction Network and Determination of Hub Genes by Mutated Genes

**FIGURE 3:**
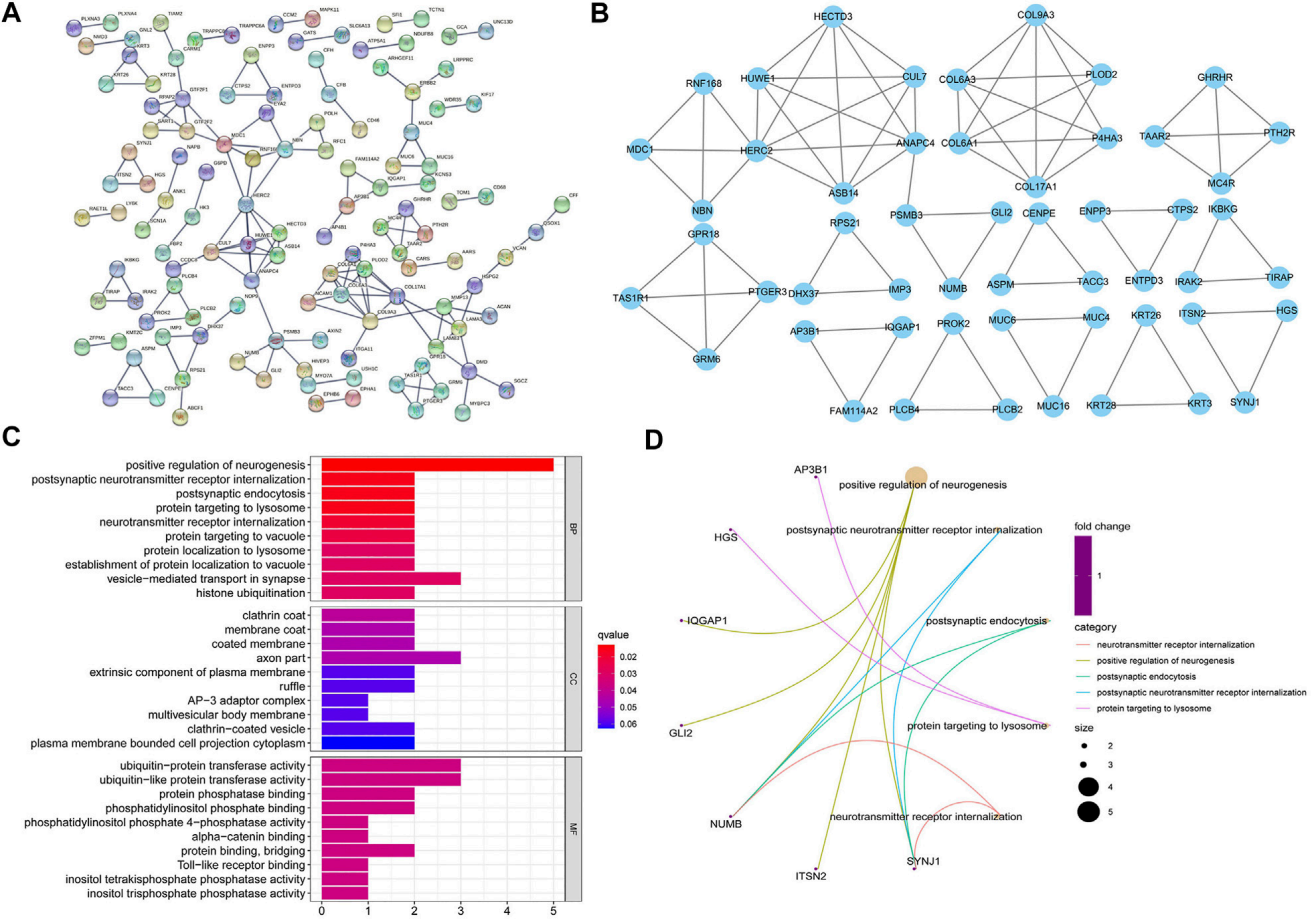
Using the protein interaction network, we could determine a protein interaction relationship between different genes which participate in biological signal transmission, gene expression regulation, and other processes. To determine which genes were in the hub position in the regulatory network, we screened the hub genes using the Molecular Complex Detection (MCODE) plug-in. The obtained hub genes were further subjected to a gene ontology (GO) enrichment analysis to observe the hub genes' primary enriched functions. **(A)** The protein-protein interactions (PPIs) of mutated single nucleotide polymorphism (SNP) genes were analyzed using the String database, and the criteria for construction were a combined score >0.9. **(B)** The most significant module in the PPI network was obtained using the MCODE plug-in for the Cytoscape 3.7.2 online software, and the criteria were obtained as follows: degree cut-off = 2, node score cut-off = 0.2, K-core = 2, Max depth = 100. **(C/D)** GO function of the 53 hub genes is mainly enriched in these three parts: biological processes (BPs), molecular functions (MFs) and cellular components (CCs) (*p* < 0.05).

The 391 mutated genes of SNPs in PSS and RSS were shown in the PPI network using String software (Figure 3A), and 53 hub mutated genes of SNPs in PSS and RSS were found by MCODE (Cytoscape plug-in) (Figure 3B). The GO analysis showed that 53 variant genes of SNPs in PSS and RSS were mainly concentrated in “positive regulation of neurogenesis,” “vesicle−mediated transport in synapse” (categorized as BPs), “axon part” (categorized as CCs), and “ubiquitin−protein transferase activity” (categorized as MFs) (Figures 3C,D). The KEGG analysis found that 53 hub mutated genes were not statistically significant (*p*-value > 0.05). We, therefore, concluded that the genes from which we obtained the mutations were of great relevance for neural differentiation and could help mediate transport.

### Survival Analysis of Hub Genes

**FIGURE 4:**
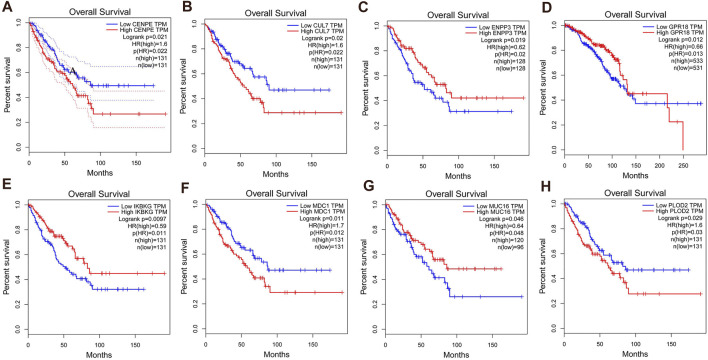
The obtained 53 hub genes were further screened according to overall survival (OS), and eight hub genes were found to be highly expressed in synovial sarcoma tissues. The threshold for prognostic survival significance was a *p-*value < 0.05. **(A)** CENPE; **(B)** CUL7; **(C)** ENPP3; **(D)** GPR18; **(E)** IKBKG; **(F)** MDC1; **(G)** MUC16; **(H)** PLOD2.

Next, we assessed OS of 53 variant hub genes of SNPs in PRSSs by the GEPIA database. There were eight hub variant genes significant for OS analysis (*CENPE, CUL7, ENPP3, GPR18, IKBKG, MDC1, MUC16,* and *PLOD2*). The result showed that all eight variant hub genes were highly expressed in SS tissues (*p* < 0.05; Figure 4). It is suggested that these eight most significant mutant hub genes may be potential markers for PSS and RSS. Basic information regarding these eight hub genes is listed in Table 2.

**TABLE 2:** The basic information of hub genes of SNPs.

SNPs Name	*p* value	Chr	MapInfo	Alleles	Transcripts	Genes	Location	Mutations	miRNA	circRNA
exm416631	0.001946	4	104066461	[A/G]	NM_001813	CENPE	EXON	Missense_F1535L	hsa-let-7a-3p	hsa_circ_0070557
									hsa-let-7b-3p	hsa_circ_0070558
									hsa-let-7f-1-3p	
exm578521	0.006285	6	131962646	[A/G]	NM_005021	ENPP3	EXON	Missense_G44R	hsa-let-7f-2-3p	None
exm1076404	0.009823	13	99907619	[T/C]	NM_001144072	GPR18	EXON	Silent	hsa-mir-1244	None
									hsa-let-7f-2-3p	
exm2248707	0.004427	X	153774337	[A/C]	NM_001042351	IKBKG	EXON	Missense_V12L	None	None
								Missense_V42L		
exm547999	0.002469	6	43008764	[T/C]	NM_014780	CUL7	EXON	Missense_R1232Q	None	None
					NM_001168370			Missense_R1316Q		
exm528596	0.008041	6	30680111	[T/C]	NM_014641	MDC1	EXON	Missense_I536M	hsa-mir-1197	None
									hsa-mir-124-3p	
									hsa-mir-1249-5p	
									hsa-mir-1253	
exm1421643	0.009823	19	9073888	[T/C]	NM_024690	MUC16	EXON	Missense_A4520T	None	None
exm356896	0.005012	3	145809622	[A/G]	NM_182943	PLOD2	EXON	Missense_C282R	hsa-mir-1271-5p	None
					NM_000935					

### Construction of the ceRNA Network Through Mutated Hub Genes

**FIGURE 5:**
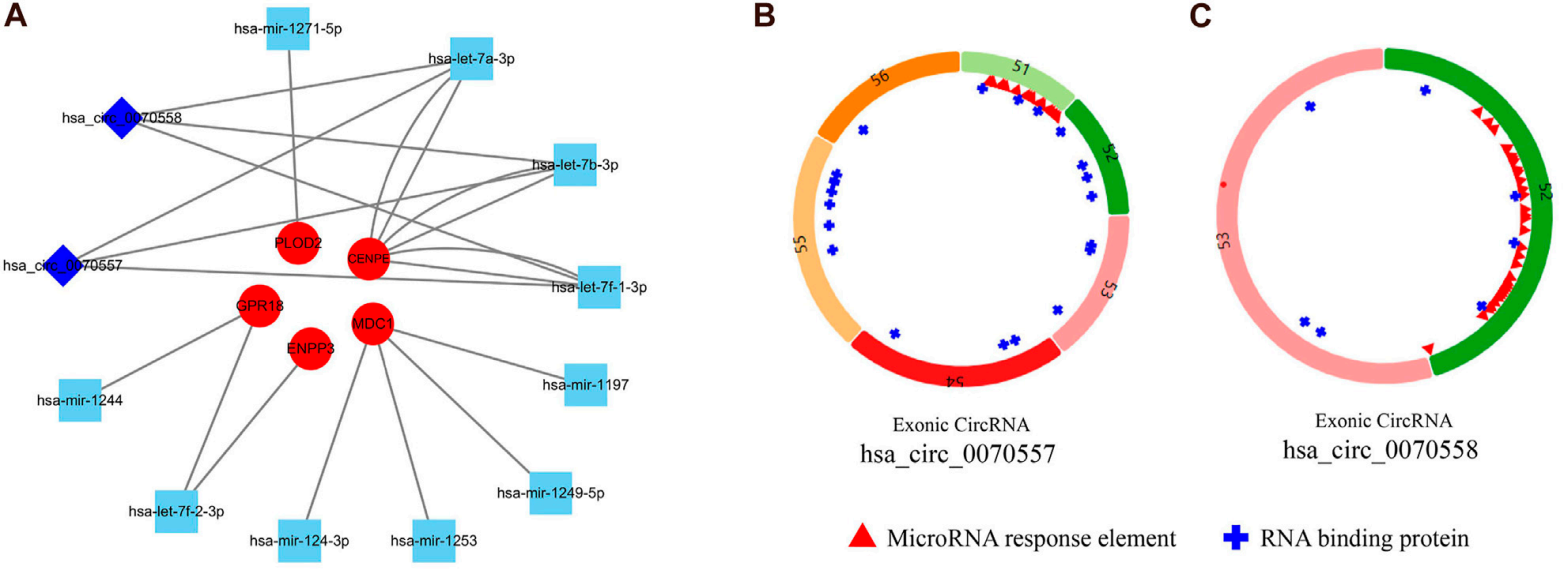
To investigate the regulatory mechanism of synovial sarcoma, we constructed a circulatoryRNA-microRNA-messengerRNA (circRNA-miRNA-mRNA) (competing endogenous RNA) network using circRNA-miRNA pairs and miRNA-mRNA pairs with Cytoscape 3.7.2. **(A)** Squares represent circRNA, diamonds represent miRNA, and circles represent mRNA. **(B)** Basic information regarding two circRNAs (has_circ_0070557 and has_circ_0070558).

In order to better understand the relationship among circRNA, miRNA, and mRNA, a ceRNA network was constructed with the SNPs of eight mutated genes (CENPE, CUL7, ENPP3, GPR18, IKBKG, MDC1, MUC16, and PLOD2). Then the corresponding miRNAs were found in two databases (miRBD and TargetScan) through the mutated hub genes. Five mutated hub genes (*CENPE, ENPP3, GPR18, MDC1,* and *PLOD2*) were found to target miRNAs (hsa-let-7a-3p, hsa-let-7b-3p, hsa-let-7f-1-3p, hsa-let-7f-2-3p, hsa-mir-1244, hsa-mir-1197, hsa-mir-124-3p, hsa-mir-1249-5p, hsa-mir-1253, and hsa-mir-1271-5p) in these two databases. The targeted circRNAs (hsa_circ_0070557 and hsa_circ_0070558) were found in three databases (Circbank, CircInteractome, StarBase) with miRNA. Finally, we used two circRNA nodes, 10 miRNA nodes, and five mutated hub genes nodes in Cytoscape 3.7.2 to form the ceRNA network (Figure 5A). Therefore, we predicted that five hub mutated genes (CENPE, ENPP3, GPR18, MDC1, and PLOD2) and two circRNAs (hsa_circ_0070557 and hsa_circ_0070558) might be suitable as potential markers for SS. The basic information regarding the circRNAs is shown in Figure 5B.

## Discussion

SS is an aggressive malignancy with biphasic differentiated sarcoma, prone to local recurrence and distant metastasis in the advanced stage (Przybyl et al., 2014). Local recurrence occurs after an average of 3.6 years and metastasis after an average of 5.7 years, and the lung is the most common site of metastasis (Krieg et al., 2011); therefore, the prognosis is poor. The origin of SS is uncertain, and studies have shown that it is derived from mesenchymal stem cells (De Logu et al., 2020). However, recent studies have suggested that neural, myogenic, or multipotent mesenchymal stem cells are considered to be the likely cellular origin (De Logu et al., 2020). It has been shown that the gene expression profile of SS is closely related to malignant peripheral nerve sheath tumors and that neural tissue-associated genes are expressed in SS tissues (Ishibe et al., 2008). Our results also showed that SS may be closely associated with neural differentiation according to the GO analysis. Histologically, SSs can be divided into monophasic, biphasic, and epithelioid types, which can present necrosis and bizarre mitoses (Soule, 1986; El Beaino et al., 2017). The morphology and clinical symptoms of SS overlap with those of various other tumors, so its diagnosis and treatment remain challenging (Thway and Fisher, 2014). Therefore, novel prognostic and predictive biomarkers, as well as novel therapeutic targets for SSs, are urgently needed.

In the era of targeted therapy, it is increasingly important to identify clinically actionable genetic alterations in cancer, and the number of these alterations is continuously increasing (Corless and Spellman, 2012). Therefore, high-throughput sequencing technologies are emerging as an essential resource for deciphering the genotype underlying a given phenotype in clinical diagnostics, cancer research, and molecularly targeted therapies for various cancers (Song et al., 2014). Many studies have found that DNA or RNA extraction is mainly performed through fresh freezing (FF), particularly for FFPE (Morris et al., 2021). FFPE tissue is the gold standard for pathological tissue preservation and represents the largest collection of patient material; its most significant advantage is its ability to be preserved long term. FFPE tissue samples provide a valuable sample resource and reduce or eliminate the need for painstaking collection and storage of cryopreserved clinical samples (Flores Bueso et al., 2020). However, the main drawback of FFPE tissue is that nucleic acid extraction is difficult owing to the need for paraffin removal with the associated loss of DNA content. Schweiger et al. (Schweiger et al., 2009) found that the decline in sequence quality was negligible in FFPE samples collected over a decade by comparing FF in whole-genome and exome sequencing. Therefore, the results suggested that reliable DNA/RNA can be obtained from FFPE tissues (Van Allen et al., 2014).

In the past few years, an increasing number of studies have shown that circRNA is much more stable than linear transcripts, mainly owing to its inability to be degraded by exonucleases, and thus this feature is thought to contribute to its effectiveness as an RNA sponge (Hansen et al., 2013). Although circRNA’s use as a sponge remains controversial, the role of circRNA as an RNA sponge is increasingly confirmed and recognized. Hansen et al. have experimentally demonstrated that Sry-derived circRNA has been found to act as a sponge for miR-138 (Hansen et al., 2013). Studies have shown that circRNA and miRNA can also serve as promising diagnostic markers and therapeutic targets and that circRNAs and miRNAs are closely related to diseases, such as cancer. Moreover, the circRNA-miRNA-mutated gene axis can be analyzed using bioinformatics to study disease pathogenesis and predict meaningful DEcircRNAs (Liang et al., 2020; Ruan et al., 2020). Zhang et al. (2018) found that obtaining circRNAs microarray analysis from FFPE samples shows comparable results to reports based on fresh frozen samples. Therefore, FFPE specimens are a good alternative to fresh frozen tissue, especially when fresh frozen specimens are limited. Bai et al. (2020) have shown that circRNA and genes were identified by the regulatory network of ceRNA, and the high expression of mutant genes was negatively correlated with OS in clear cell renal cell carcinoma (ccRCC). The potential pathogenesis of the regulatory network among circRNA/miRNA/mRNA provides some potential therapeutic options for ccRCC. Therefore, it is necessary to study the regulatory mechanisms of circRNA and hub genes, to improve the diagnosis and treatment of SS.

In investigating the potential hub genes, miRNA, and circRNA associated with PRSSs, we found eight variant hub genes (*CENPE, CUL7, ENPP3, GPR18, IKBKG, MDC1, MUC16,* and *PLOD2*), which were found to be significant on the OS analysis in SS. Some studies have shown that these mutated genes may serve as biomarkers and therapeutic targets and are related to tumor metastasis, recurrence, and prognosis. Li and Shan et al. found that CENPE can serve as a new target for the diagnosis and prognosis of rhabdomyosarcoma (Li et al., 2019) and ccRCC (Shan et al., 2019), and that it can promote the metastasis of lung cancer, and Horning et al. found that CENPE was up-regulated in prostate cancer and related to recurrent prostate cancer through RNA-sequencing data analysis.

There is increasing evidence that *CUL7* is a carcinogenic gene that can promote tumorigenesis by activating caspase-8 ubiquitination (Kong et al., 2019) and the nuclear factor-κB (Xu et al., 2020) pathway. Studies have found that *CUL7* is highly expressed in breast, lung, hepatocellular, pancreatic, ovarian, and other malignancies, thus potentially making it a novel anticancer target (Li et al., 2021). Further, some studies have shown that *ENPP3* is highly expressed in ccRCC (Moek et al., 2017). A phase I trial has been conducted with an anti-*ENPP3* antibody-drug conjugate to treat advanced refractory renal cancer (Doñate et al., 2016). Qin et al. showed that G protein-coupled receptors (GPCRs) are closely related to the occurrence and metastasis of human tumors and that *GPR18* GPCRs may be considered potential novel anticancer targets in metastatic melanoma (Qin et al., 2011). Eisinger-Mathason et al. (2013) found that the inhibition of *PLOD* enzyme activity can inhibit sarcoma metastasis, so *PLOD* is a new therapeutic target for sarcoma. Some studies have shown that IKBKG (Kang et al., 2020), MUC16 (Akahane et al., 2020) and MDC1 (Wang et al., 2018) are all related to the recurrence of breast cancer, and studies have found that MUC16 is highly expressed in most epithelial ovarian cancers and is related to the recurrence of ovarian cancer (Giannakouros et al., 2015). Therefore, MUC16 is a novel and promising tumor-associated antigen, providing an excellent target for immunotherapy, and a phase I clinical trial has been carried out (Koneru et al., 2015).

Genes can be targeted to identify upstream miRNAs. A total of 10 miRNAs were identified in this study, with studies finding hsa-let-7f-2-3p to be associated with bladder cancer progression (He et al., 2018), hsa-mir-1244 (Li et al., 2017) and hsa-mir-1197 (Yang et al., 2020) to be associated with lung cancer, and hsa-mir-1253 (Wu et al., 2020) to be associated with prostate cancer, and other miRNAs not found to be associated with cancer. It has been established that circRNA can function as “sponges” to recruit the corresponding miRNAs by interacting with miRNA binding sites, thereby playing an indirect role in regulating gene expression (Bai et al., 2020).

In this study, two circRNAs (hsa_circ_0070557 and hsa_circ_0070558) were identified, and both have yet to be reported. Therefore, the regulatory mechanism of these hub genes-miRNAs-circRNAs in SS remains unclear. Sun et al. (Zhengwang et al., 2021) analyzed transcriptomic changes of SS in differential expression, alternative splicing, gene fusion and circRNA by whole RNA sequencing in SS tissues compared with normal tissues, and found that these DEcircRNAs might act as miRNA sponges to regulate the expression of a large number of differentially expressed and alternatively spliced genes. Interestingly, we screened potential the genes that corresponded to the different genetic and SNPs altertions, by comparing PSS with RSS samples in our study. All of these studies may contribute to further understanding of the molecular genetic changes and construct gene-miRNAs-circRNAs network of SS.

Owing to the low incidence of SSs, paired PSS and RSS are particularly valuable for genome-wide association research, even though the small sample size is a limitation of this study. Therefore, our results need further validation, and the regulation mechanism of ceRNA in PSS and RSS should be further investigated regarding its significance in the diagnosis and prognosis of SS. We did not compare them with normal tissues, but Sun et al. reported the relationship between the transcriptome maps of synovial sarcomas and normal tissues, this is the difference between our study and Sun et al. Although there is some loss of DNA/RNA from the sample owing to formalin fixation, the results of this study are still reliable, and FFPE tissues are suitable for the relevant microarray analysis (He et al., 2016).

## Conclusion

The clinical application of genomics is becoming highly extensive. Herein, we analyzed genome-wide SNPs in both primary and recurrent SSs. In our study, we found that the functional annotation of SNP gene mutations is related to biological adhesion, plasma membranes, and purine nucleoside binding. These eight hub genes we screened could serve as potential targets for cancer therapy. Perticurlarly for CENPE, it can be used as a novel target for sarcoma diagnosis and prognosis. And we obtained through database that CENPE interacts with hsa-let-7a/b-3p and hsa-let-7f-1-3p family and can target hsa_circ_0070557 and hsa_circ_0070558. Therefore, this may be a potential circRNA-miRNA-mRNA regulatory network, which provides a new idea for the pathogenesis of SS and is worthy of further study on potential therapeutic targets.

## Data Availability

The datasets presented in this study can be found in online repositories. The names of the repository/repositories and accession number(s) can be found below: NCBI [accession: GSE178828].
